# A comparative study of two communication models in HIV/AIDS coverage in selected Nigerian newspapers

**DOI:** 10.3402/gha.v6i0.18993

**Published:** 2013-01-30

**Authors:** Onjefu Okidu

**Affiliations:** Department of Mass Communication, Caleb University, Imota, Lagos, Nigeria

**Keywords:** HIV/AIDS, Nigerian newspapers, communication models, activity-oriented information, cognitive-oriented information, developing countries

## Abstract

The current overriding thought in HIV/AIDS communication in developing countries is the need for a shift from the cognitive model, which emphasises the decision-making of the individual, to the activity model, which emphasises the context of the individual. In spite of the acknowledged media shift from the cognitive to the activity model in some developing countries, some HIV/AIDS communication scholars have felt otherwise. It was against this background that this study examined the content of some selected Nigerian newspapers to ascertain the attention paid to HIV/AIDS cognitive and activity information. Generally, the study found that Nigerian newspapers had shifted from the cognitive to the activity model of communication in their coverage of HIV/AIDS issues. The findings of the study seem inconsistent with the theoretical argument of some scholars that insufficient attention has been paid by mass media in developing countries to the activity model of HIV/AIDS communication. It is suggested that future research replicate the study for Nigerian and other developing countries’ mass media.

In response to the overwhelming burden of new cases of HIV infections in Africa, Asia, Latin America, and the Caribbean, the UNAIDS in 1997 initiated a project to examine the application of existing communication theories/models to HIV/AIDS prevention and care in those regions. Consequently, 103 leading global researchers were invited by UNAIDS to participate in one of five consultative workshops to review the theories/models and rethink their adequacy for Africa, Asia, Latin America, and the Caribbean. Accordingly, a new communications framework for HIV/AIDS was developed to redirect focus on the cognitive approach, which emphasises individual reason, to the activity approach, which emphasises contextual factors. The redirection came up as a response to the deficiencies of the cognitive models/theories, which are narrowly focused on the individual with insignificant consideration to critical contextual factors such as economic circumstances, political climate, and cultural environment among others (1).

Thus, since 1997, most studies and research on HIV/AIDS communication have focused on the new UNAIDS communications framework. In articles within professional journals, conferences, and other fora, scholars have discussed the inadequacies of the cognitive approach. Their conclusions echoed the UNAIDS communications framework. For instance, in the 2001 Communication for Development Roundtable, which focused on HIV/AIDS communication and evaluation, held in Managua, Nicaragua, participants unanimously agreed that the activity approach provides an alternative that overcomes the increasingly obvious limitations of the cognitive approach (2).

Similarly, reports have been published that echoed the importance of contexts of behaviour and the limitations and failures of the individual-based model of HIV/AIDS prevention by international organisations like the Panos Institute in London (3). Other international organisations have also realised the limitations of the cognitive approach. In a document that re-directs its work in communication for development, the Rockefeller Foundation (4) states that, ‘there is need to move away from a focus on individual behaviours … and on to social norms, policies, culture, etc.’ Similarly, in a meeting of the African Development Forum in Addis Ababa, Ethiopia, in December 2000, national commitment to contextual factors among others was demonstrated and articulated by African leaders (5, 6). Thus, by implication, the activity approach has become a strategic imperative for HIV/AIDS communication, especially in Africa (3).

The current overriding thought in HIV/AIDS communication in the continent therefore focuses on the need to move beyond the cognitive approach, which emphasises the decision-making of the individual, to the activity approach, which emphasises the context of the individual. One central consensus that has resulted from the discussions on the need for the shift (from the cognitive to the activity approach) is a nationally driven agenda that involves the response of relevant sectors, organisations, and stakeholders (5).

Consequently, some countries in Africa, including Nigeria, have responded by drawing up HIV/AIDS communication strategies to strengthen their national response capacities to the epidemic. Out of the estimated 33.2 million people worldwide living with the virus in 2007, Nigeria has the third-largest number after India and South Africa, with an estimated figure of 2.9 million. An estimated 168,474 people in the country became newly infected in this year (7). The first National HIV/AIDS Strategic Framework in Nigeria was implemented from 2001 to 2004, while the second phase was from 2005 to 2009. At the expiration of the first phase, the National Response review report indicated significant achievements in several areas of its implementation, including the response of the mass media and generally the communication sector (8).

However, in spite of the acknowledged media responses to the shift from the cognitive to the activity model of HIV/AIDS communication in many developing countries, some scholars (9–12) have argued that a large amount of the mass media in such countries is still predominantly grounded in HIV/AIDS cognitive information approach, though the assumption appears inconclusive as there seems to exist a noticeable gap or absence of a systematic survey on the comparative attention paid to HIV/AIDS through the cognitive and activity information models. Most studies that seem to have addressed this problem generally dwell on incidences, direction, and the nature of HIV/AIDS information using selective evidence. Therefore, there is the need to address the problem through a systematic comparison of the attention accorded to the two information dissemination models (cognitive and activity) in the mass media of individual developing countries.

## Cognitive and activity models of behaviour change communication

### Cognitive models

In *Cognition and Reality* (13), Ulric summarises the nature of the concept of cognition I ‘as the activity of knowing – the acquisition, organisation and use of knowledge.’ To Severin and Tankard (14), cognition is concerned with ‘representation’ of the world that people build in their heads and how they go about building them. Cognitive models of behaviour change communication, such as Rationality-Based Theories of Risk: Single and Situated, the Health Belief Model, the Theory of Reasoned Action, Social Learning Theory and AIDs Risk Reduction Model (ARRM), which dominated the first decade of social science research into AIDS, view the individual as a transformer of information, ‘with their pre-existing conceptions acting as filters through which information is interpreted and given significance’ (15). In a nutshell, the models seek to interpret and analyse health behaviours at the individual level where intention is independent of external influences.

### Activity model

Ryder (16) defines activity as ‘the engagement of a subject toward a certain goal or objective.’ According to him, in nature, an activity is typically unmediated while in most human contexts activities are mediated through the use of culturally established instruments, including language, artefacts, and established procedures. Carrying the argument further, Engestrom (17) calls attention to the mediation role of the community and that of social structures, including the division of labour and established procedures. Vygotsky (18) agrees with this and explains further that, an individual never reacts merely directly (or merely with inborn reflects) to the environment pointing out that cultural means and artefacts mediate the relation between the human agent and the object. The idea of *Activity model* is derived from the *Activity Theory* (19), which sees human behaviour as being part of complex and continuously collectively constructed systems of activity. Activity systems have multiple determinants and are constantly changing rather than being homogenous and stable.

In everyday life, one interacts with others through artefacts – tools or sign systems such as language. The activity of the individual is not isolated but takes place within a larger cultural historical context as human activity is socially bound and not simply the sum of individual actions (17).

## Evaluation of existing knowledge

### Theoretical perspectives

A remarkable level of consensus has been reached in the extant literature on the subject of HIV/AIDS communication in developing countries. One point of agreement is the need to move away from the cognitive models of behaviour change to the activity model. The movement is premised on the notion that past failures of preventive HIV/AIDS intervention programmes in developing countries have been wrongly blamed on the individual disregarding the contextual factors that shape the behaviour and circumstances of the individual. The factors include 1) culture, 2) politics, 3) economics, 4) gender, and 5) spiritual/religion (1, 2, 9, 11, 20). However, in trying to explain the overwhelming burden of new cases of HIV/AIDS in developing countries, some scholars (9–11, 21) have noted that insufficient attention has been paid by the media to the activity model of communication. For instance, Airhibanbuwa & Webster (3) have indicated that the media have not explored the many contextual factors surrounding the HIV/AIDS epidemic. Perhaps, the most powerful role of the media is setting the appropriate agenda in the goal of HIV/AIDS prevention, care, and support, especially given that the way a problem is defined determines the way the people try to solve it. The ability of the media to convey adequate and accurate information can be highly effective and successful in creating awareness that can lead to changes in social contexts within which individuals operate, especially in developing countries.

### Empirical studies with relevance to media coverage of HIV/AIDS in developing countries

A number of studies analysing media coverage of HIV/AIDS in developing countries (22–27) have focused on varied themes. Much of this research does not focus on systematic comparison of HIV/AIDS information types. The dominant theme has been on the volume or quantity of HIV/AIDS information. In general, they (studies) argue that there has been a decrease in the quantity of HIV/AIDS information. For instance, a study in 2005 by the International Federation of Journalists (IFJ) of media contents in Cambodia, India, the Philippines, South Africa, and Zambia found a low incidence of HIV/AIDS information. It described the findings as ‘Miniscule’ (South Africa), ‘Small’ (Cambodia and the Philippines), and ‘Infrequent’ (India).

### Empirical studies with specific relevance to media coverage of HIV/AIDS in Nigeria

A number of studies on Nigeria media coverage of HIV/AIDS (9, 26, 28–30) have mainly focused on quantity, quality, and depth of information, with tangential comparisons of individual and contextual information. For instance, a study by Journalists Against AIDS (JAAIDS) between March 2002 and March 2003 indicates that newspapers in the country generate a high volume of hard news to the detriment of news features and editorials, which give contextual depths to the pandemic. Jimoh (8) lends credence to the JAAID's survey. According to him, the most common type of HIV/AIDS reporting is news (72–82%) while the least frequent types are editorials and feature articles.

### The gap

For the most part, the empirical studies in the literature have been limited to the general analysis of 1) incidences of HIV/AIDS information, 2) depth of HIV/AIDS information, 3) focus of HIV/AIDS information, and 4) nature of HIV/AIDS in the mass media. A number of studies have focused on one or more of the above, with tangential comparisons of the individual and contextual information that define the character of the epidemic, but none have systematically offered a comparison of the attention paid to HIV/AIDS cognitive and activity information.

## Reasons for selecting the newspapers studied

In general, most Nigerian newspapers cover HIV/AIDS issues. However, eight daily newspapers were purposively chosen for the study. Several reasons guided the selection of the newspapers.

First, their popularity and consistency on the newsstand justify their use. They are among the newspapers in the country that have been published consistently since their founding. This might have increased the validity of the study, as the editions of the newspaper that were sampled for the study were available. Second, with a network of national correspondents, the newspapers have earned respect for their in-depth coverage of national issues, including HIV/AIDS. This means that the newspapers cover areas where HIV/AIDS prevalence rate is both low and high. Third, the newspapers are famous for their coverage of health issues. For instance, they have been widely cited in workshops, seminars, and training sessions on health communications in the country. Fourth, the eight newspapers are among the most widely distributed in the country and by implication the most widely read. Hence, they have the capability of attracting wider feedback on national developmental issues, including health. Fifth, the newspapers are English language dailies. The selection of only English language newspapers is because they have wider readership in Nigeria than indigenous newspapers, which are limited to particular geo-ethnic areas. The readership encompasses the elites who not only influence government policies (including health) but are themselves policy-makers. The newspapers are:

1. The Guardian
2. The Punch
3. The Vanguard
4. New Nigeria
5. Nigerian Tribune
6. ThisDay
7. Daily Trust
8. Daily Champion

## Study aim and methodology

The aim of the study is to ascertain the attention accorded to HIV/AIDS cognitive and activity information in selected Nigerian newspapers. Data were obtained by content analysis of the selected newspapers over a specified period of time. Content analysis was selected because it is the most appropriate method of providing indicators of the explicit character of Nigerian media in addressing HIV/AIDS as a development problem. The study was based on two-stage sampling: first, a sample of newspapers was taken, then a sample of newspaper editions. The units of analysis were all newspaper items (news stories, feature articles, editorials, opinions, letters to the editor, photos, cartoons, advertisements, etc.). The categories for coding content related to HIV/AIDS in the newspapers were based on those developed by JAAIDS, Nigeria, (31) on the coverage of HIV/AIDS in 11 Nigerian newspapers. The JAADS categories are similar to those that Pratt et al. (32) used in their study of HIV/AIDS information in African popular magazines and medical journals. According to Stempel (33), ‘there are real advantages to using a category system that has been used in other studies.’ One of the outstanding advantages pointed out by him is that ‘validity and reliability will be of less concern.’

The content categories are:

1. Awareness/prevention
2. Treatment/care
3. Advocacy and campaign
4. Cure claims
5. Policy pronouncements
6. Litigation
7. Statistics/trends
8. Research

To maximise consistency in coding and formally assess the intercoder reliability, the coding sheet was pilot tested. Krippendorff's alpha (α) statistic was used to verify the intercoder reliability. To compute α, the SPSS macro (KALPHA) produced by Hayes and Krippendorff (34) was employed. The output from the macro-indicated Krippendorff's α for the eight newspapers ranged from 0.891 to 0.954 (*The Guardian*, 0.891; *The Punch*, 0.918; *Tribune*, 0.903; *ThisDay*, 0.927; *Trust*, 0.944; *Champion*, 0.915; *New Nigerian*, 0.936; *Vanguard*, 0.954). Krippendorff's α for all the newspapers put together was 0.928. Given these high results, the coding sheet as well as the coders were considered reliable for the study. The Statistical Package for the Social Sciences (SPSS) was employed to analyse the study data with the aid of a computer. Descriptive statistics was used to highlight the frequency and percentage of HIV/AIDS cognitive and activity information in the selected newspapers on tables. Subsequently, the SPSS Sub-program *t*-test (independent samples) was used to test the hypotheses. The data were analysed to ascertain the attention paid to HIV/AIDS cognitive-oriented information and HIV/AIDS activity-oriented information in terms of frequency, prominence, and space.

## Major findings

### HIV/AIDS information in the eight newspapers in the period studied

Coders identified a total of 779 HIV/AIDS information leading to 908 attention scores of information location and 41365.7 col.cms of information space. Table 1 summarises the number of HIV/AIDS information published, points of attention scores of information location, and the space devoted to them in column centimetres.

**Table 1:** Summary of HIV/AIDS information in the eight newspapers in the period studied

Newspapers	Number of informations	Location scores of information in points	Space occupied in col.cms
*The Guardian*	111 (14.2%)	114 (12.6%)	4234.8 (10.2%)
*The Punch*	145 (18.6%)	176 (19.4%)	6023.6 (14.6%)
*Vanguard*	77 (9.9%)	78 (8.6%)	4104.8 (9.9%)
*New Nigerian*	116 (14.9%)	131 (14.4%)	6771.9 (16.4%)
*Tribune*	62 (8%)	77 (8.5%)	4243.9 (10.3%)
*ThisDay*	119 (15.3%)	123 (13.5%)	6690.2 (16.2%)
*Daily Trust*	92 (11.8%)	150 (16.5%)	5263.5 (12.7%)
*Daily Champion*	57 (7.3%)	59 (6.5%)	4033 (9.7%)
Total	779 (100%)	908 (100%)	41365.7 (100%)

### Frequency of HIV/AIDS information

The findings show that in terms of frequency, information on HIV/AIDS activity predominate in Nigerian newspapers. The data in Table 2 show that the total number of HIV/AIDS activity-oriented information published in Nigerian newspapers was significantly more than the total number of HIV/AIDS cognitive-oriented information during the period studied. While 499 (or 64.1%) news stories were published on HIV/AIDS activity information, only 280 (or 35.9%) news stories were published on HIV/AIDS cognitive information. Apart from the *Daily Trust* newspaper, which published an equal amount of activity and cognitive information, all of the other newspapers published more activity than cognitive information.

**Table 2:** Frequency of HIV/AIDS cognitive and activity information

		Information orientation		
			
Newspapers		Activity	Cognitive	Total
*Daily Champion*	Count	48	9	57
	% within newspaper	84.2%	15.58%	100%
*Daily Trust*	Count	46	46	92
	% within newspaper	50%	50%	100%
*The Guardian*	Count	64	47	111
	% within newspaper	57.7%	42.3%	100%
*New Nigerian*	Count	75	41	116
	% within newspaper	64.7%	35.3%	100%
*The Punch*	Count	89	56	145
	% within newspaper	61.4%	38.6%	100%
*ThisDay*	Count	88	31	119
	% within newspaper	73.9%	26.1%	100%
*Tribune*	Count	46	16	62
	% within newspaper	74.2%	25.8%	100%
*Vanguard*	Count	43	34	77
	% within newspaper	55.8%	44.2%	100%
Total		499	280	779
		64.1%	35.9%	100%

T=3.07; level of sig.=0.05; df=14; T-critical=1.76; p=0.0008 (p<0.05)

### Frequency of HIV/AIDS news stories

Count within newspapers suggests that HIV/AIDS activity-oriented news stories significantly dominated HIV/AIDS cognitive-oriented news stories across all of the newspapers. As shown in Table 3, the eight newspapers devoted a total of 356 (or 70.8%) items to activity-oriented news stories, whereas a total of 147 (or 29.2%) items were devoted to cognitive-oriented news stories.

**Table 3:** Frequency of HIV/AIDS cognitive and activity news stories

		Information orientation		
			
Newspapers		Activity	Cognitive	Total
*Daily Champion*	Count	43	6	49
	% within newspaper	87.8%	12.2%	100%
*Daily Trust*	Count	32	22	54
	% within newspaper	59.3%	40.7%	100%
*The Guardian*	Count	29	18	47
	% within newspaper	61.7%	38.3%	100%
*New Nigerian*	Count	65	31	96
	% within newspaper	67.7%	32.3%	100%
*The Punch*	Count	53	26	79
	% within newspaper	67.1%	32.9%	100%
*ThisDay*	Count	72	16	88
	% within newspaper	81.8%	18.2%	100%
*Tribune*	Count	42	9	51
	% within newspaper	82.4%	17.6%	100%
*Vanguard*	Count	20	19	39
	% within newspaper	51.3%	48.7%	100%
Total		356	147	503
		70.8%	29.2%	100%

T=3.74; level of sig.=0.05; df=14; T-critical=1.76; p=0.002 (p<0.05)

### Prominence of HIV/AIDS news stories

The results of this study also suggest that, in terms of page placement, Nigerian newspapers give more prominence to HIV/AIDS activity news stories than HIV/AIDS cognitive news stories in their coverage of HIV/AIDS issues, although the difference is not statistically significant.

Table 4 shows that out of the 557 attention scores allotted to all the news stories in the content categories, activity-oriented news stories had more points than cognitive-oriented news stories: 70.6% (or 393) to 29.4% (or 164).

**Table 4:** Prominence of HIV/AIDS activity and cognitive news stories in points

	Information orientation		
		
Location	Activity	Cognitive	Total
Front page lead	0 (0%)	0 (0%)	0 (100%)
Front page	20 (50%)	20 (50%)	40 (100%)
Back page lead	0 (0%)	0 (0%)	0 (100%)
Back page	18 (85.7%)	3 (14.3%)	21 (100%)
Page two	20 (100%)	0 (0%)	20 (100%)
Other inside pages	335 (70.4%)	141 (29.6%)	476 (100%)
Total	393 (70.6%)	164 (29.4%)	557 (100%)

T=0.650; level of sig.=0.05; df=10; T-critical=1.81; p=0.530 (p>0.05)

In all of the newspapers, there was no news story on HIV/AIDS that made front and back pages lead. There were equal proportions of news stories of HIV/AIDS activity and cognitive orientations among the news stories that appeared on the front page. Activity news stories accounted for 50% (or 20), as did cognitive news stories, which also accounted for 50% (or 20). However, among the news stories which constituted the back page, page 2, and other inside pages, activity-oriented news stories had more attention scores than cognitive-oriented news stories.

### Space devoted to HIV/AIDS news stories

Analysis of the study findings further shows that HIV/AIDS activity news stories predominate in terms of space occupation. As shown in Table 5, overall, HIV/AIDS activity-oriented news stories occupy more space than HIV/AIDS cognitive-oriented news stories. Of the total 20361.7 col.cms, space news stories occupied in the period study, HIV/AIDS activity-oriented news stories accounted for 65.3% (or 13,293 col.cms) while HIV/AIDS cognitive-oriented news stories accounted for 34.7% (or 7068.7 col.cms). Thus, HIV/AIDS activity-oriented news stories occupied proportionally more space than HIV/AIDS cognitive-oriented news stories in all of the newspapers except *Vanguard*.

**Table 5:** HIV/AIDS activity and cognitive news stories space in col.cms

	Information orientation		
		
Newspapers	Activity	Cognitive	Total
*The Guardian*	1040.3 col.cms	730.2 col.cms	1770.5 col.cms
	(58.8%)	(41.2%)	(100%)
*The Punch*	1840.4 col.cms	1310.5 col.cms	3150.9 col.cms
	(58.4%)	(41.6%)	(100%)
*New Nigerian*	2850.5 col.cms	1684.9 col.cms	4535.4 col.cms
	(62.9%)	(37.1%)	(100%)
*Tribune*	1340.2 col.cms	570.3 col.cms	1910.5 col.cms
	(70.1%)	(29.9%)	(100%)
*ThisDay*	2520.4 col.cms	610.5 col.cms	3130.9 col.cms
	(80.5%)	(19.5%)	(100%)
*Daily Champion*	1880.4 col.cms	371.5 col.cms	2251.9 col.cms
	(83.5%)	(16.5%)	(100%)
*Vanguard*	600.1 col.cms	830.5 col.cms	1430.6 col.cms
	(41.9%)	(58.1%)	(100%)
*Daily Trust*	1220.7 col.cms	960.3 col.cms	2,181 col.cms
	(56%)	(44%)	(100%)
Total	13,293 col.cms	7068.7 col.cms	20361.7 col.cms
	(65.3%)	(34.7%)	(100%)

T=2.52; level of sig.=0.05; df=14; T-critical=1.76; p=0.024 (p<0.05)

### Frequency of HIV/AIDS editorials

With regard to frequency of HIV/AIDS editorials, the findings show that although Nigerian newspapers carry more HIV/AIDS activity editorials than HIV/AIDS cognitive editorials in their coverage of HIV/AIDS issues, they carry insignificant number of editorials of activity orientation compared to editorials of cognitive orientation. Four of the eight newspapers (*Daily Trust, New Nigerian, Tribune*, and *Daily Champion*) did not publish HIV/AIDS editorials at all during the period studied (Table 6). The remaining four (*The Guardian, The Punch, ThisDay*, and *Vanguard*) with the exception of *The Punch* paid more attention to HIV/AIDS activity editorials than to HIV/AIDS cognitive editorials. The total number of HIV/AIDS activity editorials was seven (or 63.6%) while the total number of HIV/AIDS cognitive editorials was four (or 3.4%).

**Table 6:** Frequency of HIV/AIDS cognitive and activity editorials

		Information orientation		
			
Newspapers		Activity	Cognitive	Total
*The Guardian*	Count	5	3	8
	% within newspaper	62.5%	37.5%	100%
*The Punch*	Count	0	1	1
	% within newspaper	0%	100%	100%
*ThisDay*	Count	1	0	1
	% within newspaper	100%	0%	100%
*Vanguard*	Count	1	0	1
	% within newspaper	100%	0%	100%
*Daily Trust*	Count	0	0	0
	% within newspaper	0%	0%	0%
*New Nigerian*	Count	0	0	0
	% within newspaper	0%	0%	0%
*Tribune*	Count	0	0	0
	% within newspaper	0%	0%	0%
*Daily Champion*	Count	0	0	0
	% within newspaper	0%	0%	0%
Total		7	4	11
		63.6%	36.4%	100%

T=0.52; level of sig.= 0.05; df=14; T-critical=1.76; p=0.61 (p>0.05)

### Frequency of HIV/AIDS feature articles

The study findings also show that although Nigerian newspapers pay more attention to HIV/AIDS activity feature articles than HIV/AIDS cognitive feature articles in terms of frequency in their coverage of HIV/AIDS issues, they pay insignificant attention to HIV/AIDS feature articles of activity orientation compared to feature articles of cognitive orientation. Table 7 shows that HIV/AIDS activity feature articles dominated HIV/AIDS cognitive feature articles. Except for *ThisDay* and *Tribune* which had more HIV/AIDS cognitive feature articles, the remaining newspapers had more HIV/AIDS activity feature articles. Overall, the proportions for HIV/AIDS activity feature articles and HIV/AIDS cognitive feature articles were 55.6% and 44.4%, respectively.

**Table 7:** Frequency of HIV/AIDS cognitive and activity feature articles

		Information orientation		
			
Newspapers		Activity	Cognitive	Total
*Daily Champion*	Count	4	2	6
	% within news paper	66.7%	33.3%	100%
*Daily Trust*	Count	5	2	7
	% within newspaper	71.4%	28.6%	100%
*The Guardian*	Count	22	17	39
	% within newspaper	56.4%	43.6%	100%
*New Nigerian*	Count	3	2	5
	% within newspaper	60.0%	40.0%	100%
*The Punch*	Count	21	12	33
	% within newspaper	63.6%	36.4%	100%
*ThisDay*	Count	7	11	18
	% within newspaper	38.9%	61.1%	100%
*Tribune*	Count	3	7	10
	% within newspaper	30.0%	70.0%	100%
*Vanguard*	Count	9	6	15
	% within newspaper	60.0%	40.0%	100%
Total		74	59	133
		55.6%	44.4%	100%

T=0.55; level of sig.=0.05; df=14; T-critical=1.76; p=0.59 (p>0.05)

### Space devoted to HIV/AIDS feature articles

In terms of space allocation, the findings show that Nigerian newspapers devote insignificantly more space to HIV/AIDS feature articles of activity orientation than HIV/AIDS feature articles of cognitive orientation in their coverage of HIV/AIDS issues. As shown in Table 8, the ratio of space in col.cms occupied by HIV/AIDS activity-oriented feature articles to HIV/AIDS cognitive-oriented feature articles in all the newspapers put together indicates that HIV/AIDS activity-oriented feature articles occupied more space than HIV/AIDS cognitive feature articles. During the period studied, Nigerian newspapers devoted a space of 8582.4 col.cms (or 55.2%) to activity-oriented feature articles and a space of 6961.7 col.cms (or 44.8%) to cognitive-oriented feature articles.

**Table 8:** HIV/AIDS cognitive and activity feature articles space in col.cms

	Information orientation		
		
Newspapers	Activity	Cognitive	Total
*The Guardian*	2640.5 col.cms	2,040 col.cms	4680.5 col.cms
	(56.4%)	(43.6%)	(100%)
*The Punch*	2,040 col.cms	2160.4 col.cms	4200.4 col.cms
	(48.6%)	(51.4%)	(100%)
*New Nigerian*	240 col.cms	300.6 col.cms	540.6 col.cms
	(44.4%)	(55.6%)	(100%)
*Tribune*	600 col.cms	420.7 col.cms	1020.7 col.cms
	(58.8%)	(41.2%)	(100%)
*ThisDay*	1320.2 col.cms	780 col.cms	2100.2 col.cms
	(62.9%)	(37.1%)	(100%)
*Daily Champion*	240.3 col.cms	360 col.cms	600.3 col.cms
	(40%)	(60%)	(100%)
*Vanguard*	1,080 col.cms	720 col.cms	1800.8 col.cms
	(60%)	(40%)	(100%)
*Daily Trust*	420.6 col.cms	180 col.cms	600.6 col.cms
	(70%)	(30%)	(100%)
Total	8582.4 col.cms	6961.7 col.cms	15544.1 col.cms
	(55.2%)	(44.8%)	(100%)

T=0.484; level of sig.=0.05; df=14; T-critical=1.76; p=0.636 (p<0.05)

### Frequency of HIV/AIDS advertisement messages

With regard to frequency of advertisement messages, the study findings show that although Nigerian newspapers carry more HIV/AIDS activity advertisement messages than HIV/AIDS cognitive advertisement messages in their coverage of HIV/AIDS issues, the difference is not statistically significant. The distribution of data in Table 9 shows that, generally, HIV/AIDS activity advertisement messages were higher than HIV/AIDS cognitive advertisement messages. However, HIV/AIDS cognitive advertisement messages were higher than HIV/AIDS activity advertisement messages in *Daily Trust* and *The Punch* newspapers.

**Table 9:** Frequency of HIV/AIDS activity and cognitive advertisement messages

		Information orientation		
			
Newspapers		Activity	Cognitive	Total
*Daily Champion*	Count	1	0	1
	% within newspaper	100%	0%	100%
*Daily Trust*	Count	5	16	21
	% within newspaper	23.8%	76.2%	100%
*The Guardian*	Count	3	0	3
	% within newspaper	100%	0%	100%
*New Nigerian*	Count	2	0	2
	% within newspaper	100%	0%	100%
*The Punch*	Count	4	5	9
	% within newspaper	44.4%	55.6%	100%
*ThisDay*	Count	7	0	7
	% within newspaper	100%	0%	100%
*Tribune*	Count	1	0	1
	% within newspaper	100.0%	0.0%	100.0%
*Vanguard*	Count	4	0	4
	% within newspaper	100%	0%	100%
Total		27	21	48
		56.3%	43.7%	100%

T=0.35; level of sig.=0.05; df=14; T-critical=1.76; p=0.73 (p>0.05)

## Discussion

Overall, the findings of this study seem consistent with the study assumption that given the strategic role the Nigerian media have been playing in setting contextual agenda since the country engaged in multi-sectoral and community-based response to HIV/AIDS prevention, treatment, care, and support, activity information would be accorded more attention than cognitive information in the local newspapers. The significant coverage in the frequency of HIV/AIDS activity-oriented information as identified in Nigerian newspapers confirms that HIV/AIDS contextual issues are high on their agenda. However, the findings seem inconsistent with the view of some scholars (8–10, 11) that insufficient attention has been paid by the mass media in developing countries to the activity model of HIV/AIDS communication. On the contrary, the findings of this study suggest that the Nigerian mass media have shifted from individual-level models of preventive HIV/AIDS communication to more multi-level, culturally sensitive, and contextually relevant explanations and interventions. The shift in focus of Nigerian newspapers from the cognitive model to the activity model of HIV/AIDS communication further shows that the Nigerian mass media in their expanded roles are capable of providing national and international models of strategic responses to diseases. Therefore, the overwhelming burden of new cases of HIV/AIDS in Nigeria cannot be attributed to diminished media coverage of contextual elements.

However, the insignificant difference in frequency between HIV/AIDS activity-oriented editorials as well as feature articles and HIV/AIDS cognitive-oriented items in the same category suggests that the activity information in Nigerian newspapers is not in-depth. One would have expected Nigerian newspapers to have published significantly more HIV/AIDS activity-oriented editorials and feature articles than HIV/AIDS cognitive-oriented items of the same orientation. This is because these items, more than the straight news, have the capacity to lay bare the way in which HIV/AIDS exacerbates social prejudices, economic inequalities, discriminatory practices and political injustices. In addition, they (editorials and feature articles) have the capacity to stimulate debate and foster a social environment in which AIDS is addressed in the spirit of openness and communality.

It can be observed that straight news stories dominated the HIV/AIDS information items studied (see Table 3). It is also noteworthy that there is a relative lack of editorials, feature articles, as well as advertisement messages. Hence, the insignificant coverage of activity information of editorials, features, and advertisement orientation may suggest a feeling of ambivalence occasioned by a lack of skill and knowledge on the part of editorial and feature writers in the various newspapers studied. This shows that the Nigerian media HIV/AIDS agenda is not set by individual media organisations but is driven largely by the agenda of those in other stakeholders, particularly the government and civil society activists. Furthermore, it may be that the Nigerian media prior to the 1997 new UNAIDS communication framework were engrossed in the cognitive model so much that the approach still impacts on them (Nigerian mass media). Thus, Nigerian newspapers have yet to do full justice to the contextual elements that drive the epidemic in a vibrant and open manner, perhaps because of their desire to ‘sell newspapers’ rather than responding to a challenge demanding intervention from all sectors.

The insignificant difference in prominence between HIV/AIDS activity-oriented news stories and HIV/AIDS cognitive-oriented news stories is suggestive of the editorial value Nigerian newspapers attach to HIV/AIDS activity-oriented new stories in particular and HIV/AIDS stories in general. As pointed out earlier, it may be that Nigerian newspapers were still withdrawing from the cognitive model or better still, it could be as a result of the unimportant editorial value they attach to HIV/AIDS information generally. It is noteworthy that none of the newspapers sampled used an HIV/AIDS story as front or back page lead. Furthermore, both HIV/AIDS activity and cognitive stories have low attention scores on the front and back pages (see Table 5). A number of reasons could be adduced for it. It could be that the urge to sell had conditioned the perception of the newspapers towards considering HIV/AIDS stories as not being sensational enough. It could also be a manifestation of the natural inclination of the newspapers to follow occurrences that had ‘greater’ social impact.

For page placement of information, the insignificant difference between HIV/AIDS activity-oriented feature articles and HIV/AIDS cognitive-oriented feature articles is indicative of the social significance Nigerian newspapers attach to HIV/AIDS activity-oriented feature articles. Perhaps, this is because print space dedicated to one topic invariably displaces another and editors might not have the ‘see value’ in dedicating scarce and valuable column centimetres to activity feature articles. In other words, activity feature articles were not given the required contextual depth. This seems consistent with the argument of Projecthope (31) that HIV/AIDS information in the mass media of developing countries does not go beyond the simplistic, episodic, and superficial levels to explore the contextual factors driving the epidemic in detail.

For a country that has the third largest HIV infection rate in the world, one would have expected the mass media to deliberately play an instrumental and a more active role in the battle against the disease by setting the agenda for in-depth contextual discourse. Nigerian newspapers have the greatest challenge by becoming more responsive.

## Conclusion

Generally, this study shows that Nigerian newspapers have shifted from the cognitive to the activity model of HIV/AIDS communication. Their gatekeeping, agenda setting, and agenda-building roles underscore the likelihood that the mass media in other developing countries may not be grounded in the cognitive model of HIV/AIDS communication, as observed by some scholars. They may be involved in HIV/AIDS communication using the activity model. It is therefore suggested that this study be replicated for the mass media of other developing countries, the Nigerian radio, television, magazines, and weekly/monthly newspapers to ascertain their level of response to the global call for a shift from the cognitive model to the activity model of HIV/AIDS communication.

## References

[1] UNAIDS/PENNSTATE (2009). Communications framework for HIV/AIDS.

[2] UNFPA (2002). Communication for development: focus on HIV/AIDS communication and evaluation.

[3] Airhihenbuwa CO, Webster J Culture and African contexts of HIV/AIDS: prevention, care and support. http://scholar.goggle.com/scholar.

[4] Rockfeller Foundation (1999). Communication for social change.

[5] Rawoo (2002). Making social science matter in the fight against HIV/AIDS.

[6] Airhihenbuwa CO, Makinwa B, Obregon R (2000). Toward a new communications framework for HIV/AIDS. J Health Commun.

[7] UNAIDS/WHO (2007). Report on the global AIDS epidemic. http://www.unaids.org.

[8] NACA (2005). Nigeria: HIV/AIDS country report.

[9] Kelly P, Parker W, Lewis G, Stones (2001). Reconceptualising behaviour change in the HIV/AIDS context. Socio-political and psychological perspectives on South Africa.

[10] Jimoh AK Gaps in HIV/AIDS reporting in Nigeria: problems, and prospects for media NGO partnership. http://gateway/n/m.nih.go.gov/meeting.

[11] Parker W Rethinking conceptual approaches to behaviour change: the importance of context. http://www.cadre.org.2a.

[12] Schoofs M (2000). The media is the message: a case study in AIDS journalism.

[13] Ulric N (1976). Cognition and reality.

[14] Severine WJ, Tankard JW (1992). Communication theories: origin, methods and uses in the mass media.

[15] Bajos N (1997). Social factors and the process of risk construction in HIV sexual transmission. AIDS Care.

[16] Ryder M What is activity theory?. http://carboncudeniver.edu/myrder/itc-data-diff.html.

[17] Engestrowm Y, Engestrom Y, Meiltinen R, Punamaki RL (1999). Activity theory and individual and social transformation. Perspective on activity theory.

[18] Vygotsky LS (1987). Collected works.

[19] Chaiklin S, Lave J (1993). Understanding practice: perspectives on activity and context.

[20] UNAIDS (2000). HIV/AIDS and Communication for behaviour and social change: programme experience, examples, and the way forward.

[21] Cullinan K (2001). The media and HIV/AIDS: a blessing and curse.

[22] Pitts M, Jackson H (1992). Press coverage of AIDS in Zimbabwe: a five-year review. AIDS Care.

[23] Seidel G (1993). The competing discourses of HIV/AIDS in Sub-Saharan Africa: discourses of rights and empowerment vs discourses of control and exclusion. Soc Sci and Med.

[24] Mchombu K, Kwame ST, Boafo CA, Arnaldo K (2000). The coverage of HIV/AIDS in Namibian media: a content analysis Study. Media and HIV/AIDS in East and Southern Africa. A resource book.

[25] IWMF & AWMC The Media's response to covering HIV/AIDS, TB and malaria in Africa. http://www.iwmf.org.

[26] IFJ Reporting HIV/AIDS in six countries in Africa and Asia. http://www.ifg.org/pdfs.

[27] Kasoma FP (1990). The Zambian press and AIDS crisis. CAEJAC Journal.

[28] JAAIDS (2003). A slow awakening: the media and HIV/AIDS epidemic in Nigeria: A scorecard.

[29] Adesemoye AO Fighting HIV/AIDS in Nigeria: the performance of the media so far, what Steps Next?. http://www.aegis.org/conference/iac/2002/f1212b.html.

[30] Projekthope There is no town without HIV/AIDS. http://www.sunnewsonline.com.

[31] Kasoma FP (1996). Content analysis of AIDS stories in Zambian media. CAEJAC Journal.

[32] Pratt BC, Pratt CA, Ha L (2002). Setting the public health Agenda on major diseases in Sub-Saharan Africa: African popular magazines and medical journals 1981–1997. J Commun.

[33] Stempel GH, Guido H, Brues W (1989). Content analysis. Research methods in mass communication.

[34] Hayes AF, Krippendorff K Answering the call for a standard reliability measure for coding data: Brief report. http://www.comm.ohio-state.edu/ahayes/spss/20programs/kalpha.htm-31k.

